# A Biomimetic DNA‐Based Membrane Gate for Protein‐Controlled Transport of Cytotoxic Drugs

**DOI:** 10.1002/ange.202011583

**Published:** 2020-11-24

**Authors:** Conor Lanphere, Patrick M. Arnott, Sioned Fôn Jones, Katarina Korlova, Stefan Howorka

**Affiliations:** ^1^ Department of Chemistry Institute of Structural and Molecular Biology University College London London WC1H 0AJ UK; ^2^ Department of Biochemical Engineering University College London London WC1E 7JE UK; ^3^ Department of Chemistry King's College London London SE1 1DB UK

**Keywords:** aptamers, biosensors, DNA structures, membrane, nanopores

## Abstract

Chemistry is ideally placed to replicate biomolecular structures with tuneable building materials. Of particular interest are molecular nanopores, which transport cargo across membranes, as in DNA sequencing. Advanced nanopores control transport in response to triggers, but this cannot be easily replicated with biogenic proteins. Here we use DNA nanotechnology to build a synthetic molecular gate that opens in response to a specific protein. The gate self‐assembles from six DNA strands to form a bilayer‐spanning pore, and a lid strand comprising a protein‐binding DNA aptamer to block the channel entrance. Addition of the trigger protein, thrombin, selectively opens the gate and enables a 330‐fold increase inw the transport rate of small‐molecule cargo. The molecular gate incorporates in delivery vesicles to controllably release enclosed cytotoxic drugs and kill eukaryotic cells. The generically designed gate may be applied in biomedicine, biosensing or for building synthetic cells.

## Graphical abstract

DNA nanotechnology can be used to rationally design a molecular gate to control the transport of small‐molecule drugs across bilayer membranes. The DNA pore with a thrombin‐binding aptamer lid opens in the presence of thrombin to increase the transport of the cytotoxic drug topotecan by 330‐fold. The nanodevice could be adapted for a range of different protein triggers with applications in biosensing, research, and biomedicine.

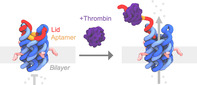

## Introduction

Creating engineered or synthetic membrane channels is of interest in science and technology. Constitutively open, barrel‐like channels have been harnessed for next‐generation portable DNA sequencing and biosensing.[^1^, ^2^, ^3^, ^4^] In this approach, individual analyte molecules pass through the channel and cause electrical signatures.[^5^, ^6^, ^7^] Advanced membrane gates with valve‐like function are also of considerable interest. Biological membrane gates specifically recognize bio‐ligands, such as proteins, and in response open or close to control transport across lipid bilayers. This advanced function could be used in biosensing approaches,^[8]^ drug delivery systems,^[9]^ or synthetic cell‐like entities.^[10]^ Adapting natural gates^[11]^ for applications outside their biological remit is, however, difficult. One hurdle is the complex conformational changes between molecular recognition and channel opening. Hence, there is demand for a simple chemical strategy to build de novo channels[^12^, ^13^, ^14^] with defined molecular recognition with an effective opening mechanism. Ideally, the synthetic functional gates would be suitable to regulate flux of bioactive substances across membranes.

Rational design with DNA is an attractive route to predictably self‐assemble structurally defined DNA nanoarchitectures.[^15^, ^16^, ^17^, ^18^, ^19^, ^20^, ^21^] DNA nanotechnology has yielded barrel‐shaped bundles of DNA duplexes that puncture membranes with tuneable channel diameters.[^22^, ^23^, ^24^, ^25^, ^26^] Versions have also been made where a DNA strand reversibly blocks the channel lumen in response to a complimentary ligand strand, or temperature to enable fluorophore transport.[^24^, ^27^] However, an unmet challenge is to rationally design advanced channels that are triggered by biological ligands to control flux of bioactive cargo.

**Figure 1:**
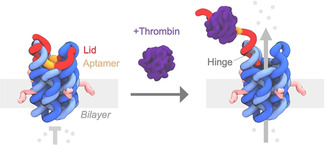
DNA‐based protein‐gated nanopore, pNP, opens upon thrombin binding allowing the transport of material. The pore's barrel is composed of six DNA strands (light and dark blue) that form six interconnected DNA duplexes arranged in a hexagonal fashion. The protein‐responsive lid (red, orange) features the thrombin‐binding aptamer (orange), which is bound to the pore via two extended docking loops, including the hinge region. In the closed state, the lid blocks the channel of pNP. Binding to thrombin (dark purple) leads to the partial dissociation of the lid and opening of pNP's channel, allowing cargo transport. The lid remains attached to the hinge region of the pore. Four cholesterol anchors (pink) insert pNP into the hydrophobic lipid bilayer membrane.

Here we use DNA nanotechnology to build an artificial protein‐regulated molecular gate for off‐on switched transport of cytotoxic drugs. The protein‐gated nanopore, pNP, is composed of six DNA oligonucleotides forming a membrane‐spanning six‐duplex nanobarrel with a 2 nm‐wide inner lumen (Figure 1, light and dark blue),^[24]^ and a 7^th^ strand, a protein‐responsive lid to regulate transport (Figure 1, red and orange). The outer dimensions of the molecular gate are approximately 13×5×5 nm.

## Results and Discussion

In pNP's closed state, the lid strand was designed to span the channel entrance by binding to three docking sites and a hinge at the top of the pore (Figure 1,left panel; Figure S1,S2).[^24^, ^27^] To function as a tuneable protein‐sensitive gate, the lid was coded with a DNA aptamer sequence (Figure 1, orange)(Figure S1,S2). Aptamers are usually composed of a single nucleic acid strand and in their folded state bind to analytes with high affinities and specificity.[^8^, ^28^, ^29^, ^30^, ^31^, ^32^, ^33^, ^34^, ^35^, ^36^, ^37^] In our protein‐gated nanopore, we used the well characterized thrombin binding aptamer (TBA) (Figure 1, orange).^[38]^ The 15‐nt long TBA sequence forms a G‐quadruplex which binds human alpha‐thrombin with a dissociation constant, *K*_d_, between 100 and 200 nm.[^38^, ^39^] The TBA sequence and another part of the lid span the pore entrance in the closed pNP (Figure 1, left panel)

**Figure 2:**
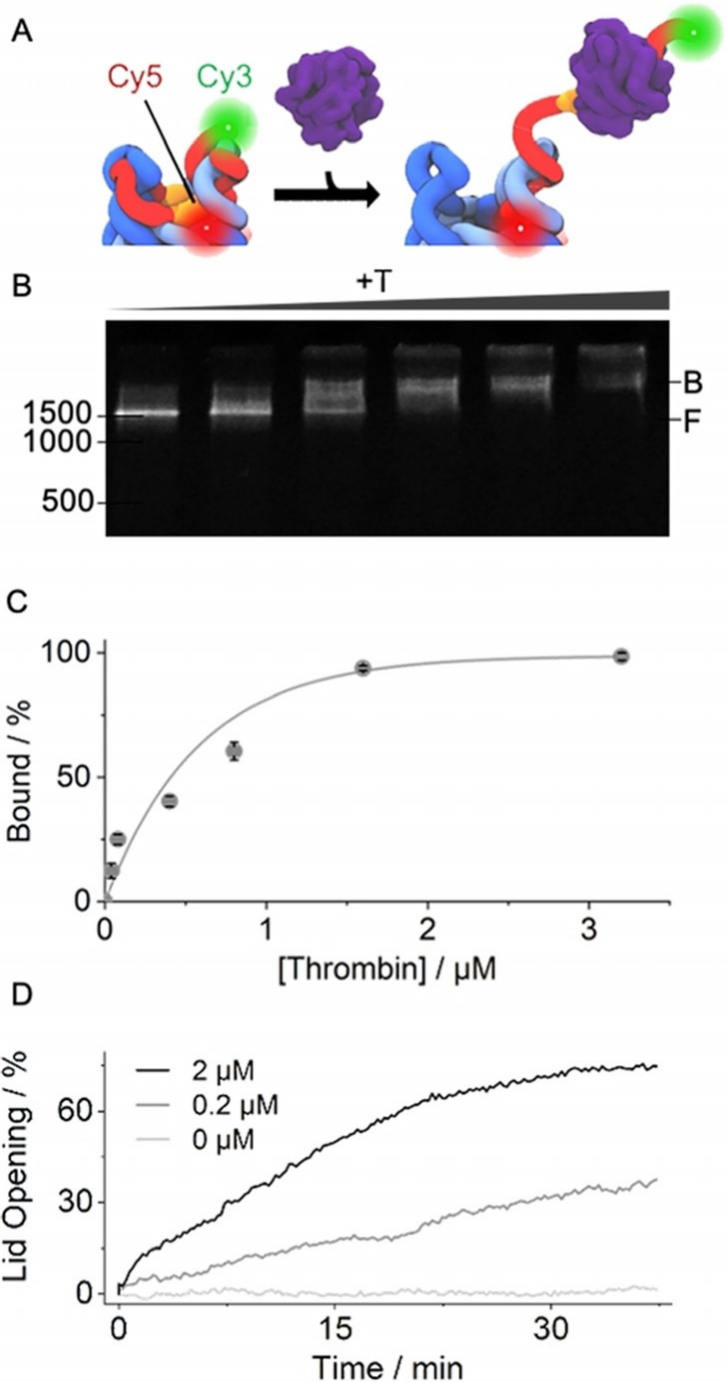
Thrombin binding actuates pNP lid‐opening. A) 3D scheme of pNP's top with TBA sequence (orange) in the lid (red) which carries a Cy3 fluorescence donor while the Cy5 acceptor is linked to the pore. Thrombin‐induced lid opening separates the dyes and enhances Cy3 emission. B) Gel shift assay illustrating pNP–thrombin binding. Increasing concentrations of thrombin lead to a progressive mobility shift from the free pNP (F) to the bound pNP–thrombin complex (B). The position and bp length of the dsDNA markers are given at the left of the gels. C) Quantitative analysis of the gel‐shift data via plotting the normalised amount of the pNP–thrombin complex against the concentration of thrombin. The amount of the complex is derived from the gel band intensities via 1−(I_pNP_−I_background_) and normalisation to maximum binding. The derived *K*_d_ is 662±93 nm (*n*=3). D) Kinetic Cy3 fluorescence emission supports lid opening upon thrombin binding at different concentrations of thrombin of 0.2 μm (grey) and 2 μm (black) while buffer without protein (light grey) causes no change. For 100 % lid opening, a sample of pNP was incubated for 30 min at 55 °C, which is higher than the melting temperature of the lid with docking regions D_1_‐D_3_ but below that of the hinge, which tethers the lid to the pore.

For pNP opening, thrombin binds to the lid's TBA domain and partially unzips the lid (Figure 1, right panel; thrombin, purple; Figure S2). Following our rational design, lid dissociation is anticipated only at the lid docking regions (D) but not at the hinge region (H) due to different melting temperatures for the respective domains, which are D_1_=45 °C, D_2_=40 °C, D_3_=45 °C, and H=65 °C (Figure S1). The sequences of the component oligonucleotides and 2D DNA maps of pNP are summarized in Tables S1, Table S2, and Figure S1. Two variants of the protein‐gated nanopore, pNP and pNP2, were designed to probe the influence of TBA position on gate function. In pNP, the TBA sequence is located in the channel‐spanning section of the lid between docking regions D_1_ and D_2_, while in variant pNP2, the TBA sequence is located in the other channel‐spanning section between docking region D_3_ and the hinge, H (Figure 2 A, Table S1, Figure S1). All pores carry four cholesterol modifications to facilitate insertion into lipid bilayer membranes (Table S1,S2, Figure S1).

Prior to building the protein‐gated nanopore, we determined the affinity of TBA for thrombin using an electrophoretic mobility shift assay. TBA contained a 26‐nt tail to enhance staining in the assay (Table S1). A defined up‐shift of the gel band upon thrombin addition implied the formation of a complex between the aptamer and thrombin (Figure S3A). Its affinity was obtained by determining the band intensity of the complex as a function of thrombin concentration (Figure S3).^[40]^ Plotting the binding curve and applying a Langmuir fit yielded a *K*_d_ of 152±11 nm (*n*=3). The result confirmed stoichiometric binding between TBA and thrombin (Figure S3).^[38]^ The *K*_d_ value is in agreement with other published studies.[^8^, ^38^, ^39^]

The protein‐gated nanopore pNP was self‐assembled by annealing an equimolar mixture of the six pore oligonucleotides plus the lid strand containing the TBA sequence (Figure 1, Figure S1). Successful folding of pNP was confirmed by the presence of a single band in gel electrophoresis (Figure S4). The barrel without the lid migrated faster due to its smaller size and molecular weight (Figure S4). pNP2 was also assembled successfully and, as expected, ran similarly to pNP in gel electrophoresis (Figure S4).

pNP's affinity for thrombin was then probed using a gel shift assay. The assay is able to discriminate pNP from the pNP–thrombin complex due to their different migration through the gel matrix (Figure 2 B, F and B, respectively). The formation of the complex was followed by increasing the concentration of thrombin (Figure 2 B). No other faster migrating bands were detected (Figure 2 B), suggesting that pNP remains fully intact following thrombin binding (Figure 1). Complete binding was observed at a ratio of 20:1 of thrombin to pNP (Figure 2 B). The affinity of the thrombin–pNP interaction was determined by plotting the gel band intensities of the complex against the thrombin concentration (Figure 2 C). The Langmuir fit‐derived *K*_d_ was 662±93 nM. The *K*_d_ value is higher than for the isolated TBA‐thrombin and can be attributed to reduced steric accessibility of the TBA sequence in pNP (Figure 1, Figure 2 A). The same analysis route revealed that pore variant pNP2 had a two‐fold weaker *K*_d_ of 1.31±0.26 μm (*n*=3) and required a 40:1 ratio for stoichiometric binding (Figure S5). The data indicate that both lid designs yield a functional response.

The ability of thrombin to unzip the lid from pNP was monitored kinetically using fluorescence emission. pNP was equipped with a donor Cy3 dye at the 3′ end of the lid and an acceptor Cy5 dye on the barrel (Figure 2 A, Table S1,S2). In the closed state of pNP, the Cy3 emission was expected to be low due to its close proximity to Cy5 (Figure 2 A, Figure S6). In contrast, thrombin binding was anticipated to unzip the lid and increase the distance between Cy3 and Cy5 (Figure 2 A, Figure S6). The expected increase in Cy3 emission was confirmed by kinetic fluorescence measurements at 0.2 and 2 μm thrombin leading to 36 % and 75 % higher fluorescence, respectively (Figure 2 D)(0.1 μm pNP). No unzipping was observed in the absence of thrombin (Figure 2 D). pNP2 displayed similar opening kinetics (Figure S7). In addition, when the TBA sequence was absent from the lid, no gate opening was observed (Figure S8, pNP3). The reversibility of the lid opening was not tested but could be achieved by a shorter hinge region between aptamer and nanopore to allow dissociation of aptamer‐thrombin complexes, or competitive displacement of aptamer‐thrombin with free aptamer strand.

Confocal laser scanning microscopy confirmed pNP was successfully anchored to bilayer membranes. A version of the protein‐gated nanopore carrying a TAMRA dye (Figure S1, pNP^TAMRA^) was incubated with POPC giant unilamellar vesicles (GUVs). Microscopic analysis showed successful binding of the lipid‐anchored pore to bilayer membranes by the formation of a fluorescent halo around the GUV perimeter (Figure S4).

**Figure 3:**
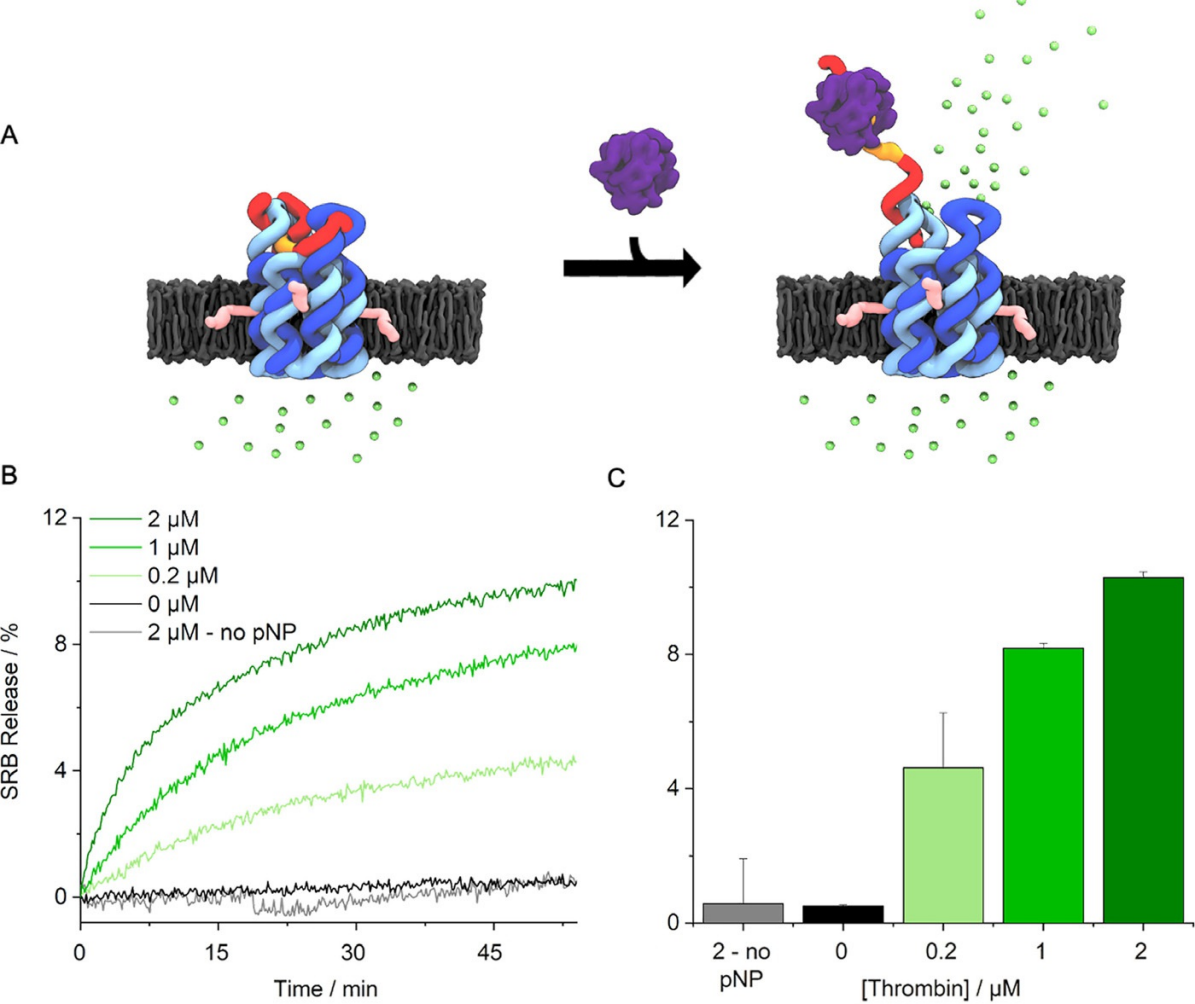
Protein‐actuated opening of pNP controls transport of molecular cargo across lipid bilayers. A) pNP is embedded in the lipid bilayer of a vesicle filled with the fluorophore sulforhodamine B (SRB, green dots). The dye is contact‐quenched at 50 mm inside the vesicle. In the closed state of pNP, the encapsulated SRB cannot traverse the membrane. Thrombin‐binding results in the partial unzipping of the lid and pore opening to release SRB into the ambient. The lower dye concentration abolishes contact‐quenching and increases fluorescence. For visual clarity, a pore inserted in the membrane in the opposite orientation is not shown. The mixed orientations can lower the release to a degree of up to 50 %. B) Kinetic traces of SRB fluorescence as a function of increasing pNP concentration. 100 % release is the total amount of fluorescence obtained upon rupturing vesicles with a detergent. C) Bar chart of net fluorescence increase, summarizing data from (B). The data represent averages and standard deviations from at least 3 independent experiments.

Controlled transport across membrane‐inserted pNP was probed with a dye flux assay (Figure 3 A). Sulforhodamine B (SRB) was encapsulated inside large unilamellar vesicles (LUVs) (Figure S9) at a high, self‐quenching concentration.^[41]^ Upon dye transport, its concentration and quenching effect decreases giving rise to a significant increase in detectable fluorescence. Addition of thrombin was expected to open pNP and thereby enable dye efflux and fluorescence emission (Figure 3 A). In line with expectations, there was no change in fluorescence over 55 min when either pNP or thrombin was absent from the assay (Figure 3 B). By contrast, emission was strongly increased upon addition of thrombin at 0.2 to 2 μm (Figure 3 B). The data demonstrate protein‐triggered opening of pNP to enable transport of the dye. Analysis of the efflux rates revealed a 330‐fold enhancement upon protein binding (Figure 3 C) from an average of 0.0039±0.010 %/min (*n*=7) at 0 μm thrombin to 1.2±0.17 %/min at 2 μm thrombin(*n*=3) (Figure 3 C). Very similar fluorescence profiles confirmed protein‐triggered opening for pNP2 (Figure S10,S11). In further support, a higher nanopore concentration led to higher release activity (Figure S12) while no release was observed when using a pore variant without the TBA sequence (Figure S13, pNP3).

**Figure 4:**
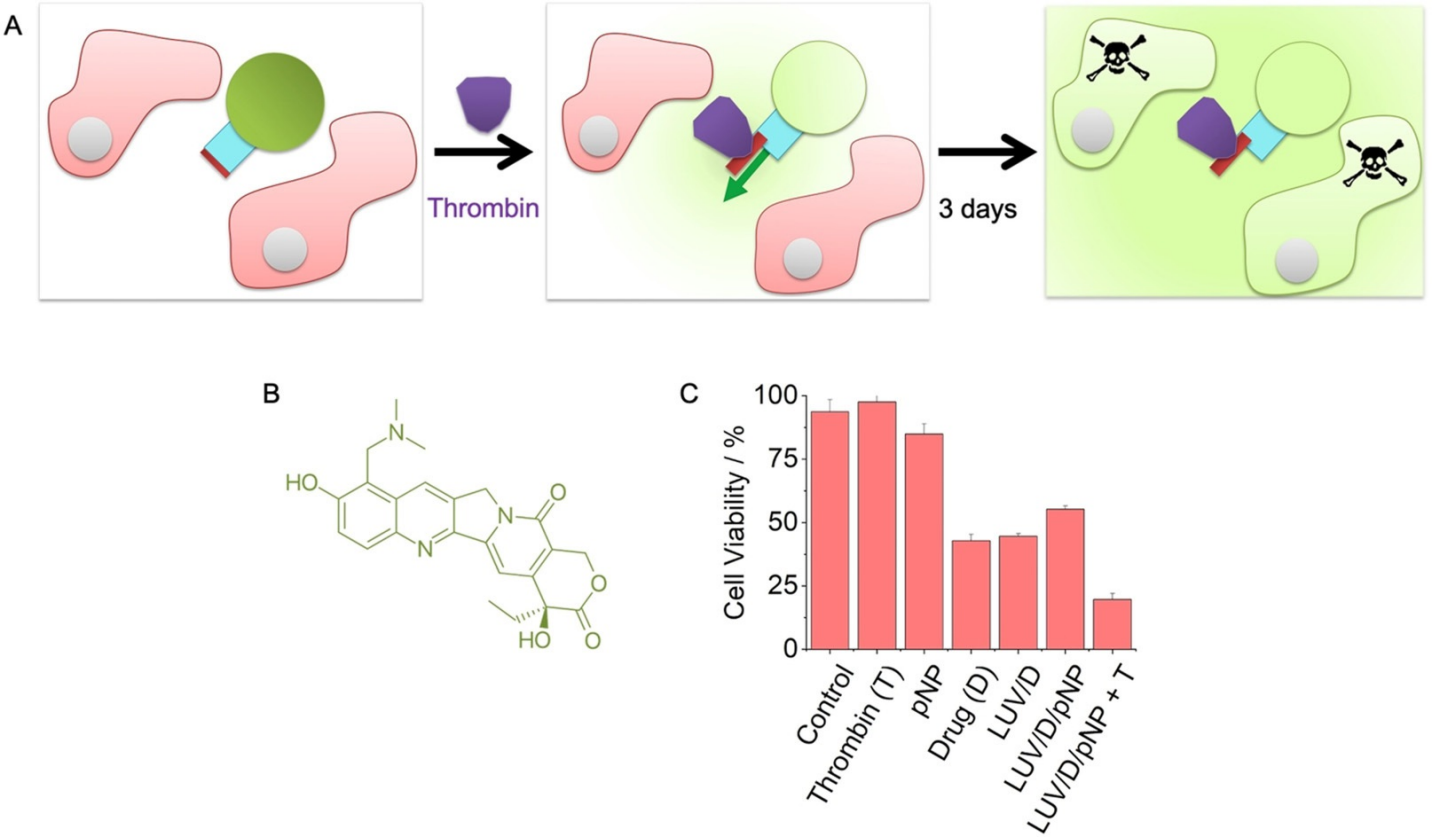
Protein‐triggered opening of pNP releases a cytotoxic drug for controlled killing of cells. A) Scheme of the assay to demonstrate the controlled killing of HeLa cells (pink). The cells are exposed to pNP‐functionalised‐membrane vesicles filled with the cytotoxic drug, topotecan (green), as well as thrombin (purple) to open pNP (blue, red), followed by incubation for 3 d to attain the cytotoxic effect of released topotecan. For visual clarity, a pore inserted in the membrane in the opposite orientation is not shown. The mixed orientations can lower the release to a degree of up to 50 %. B) Chemical structure of topotecan. C) Graph displaying the viability of HeLa cells after 3 d incubation with either thrombin, pNP, topotecan, topotecan‐filled LUVs with a lipid ratio of PC:PE (7:3), pNP‐functionalised topotecan‐filled LUVs, and the latter in combination with thrombin. The data are the means±SD collected from three independent experiments. The assay was carried out with pNP2 but is referred to pNP for reasons of simplicity and because both molecular gate variants have very similar functional properties. The cell viability was determined with the WST‐1 assay.

In the final part of our study, we investigated the use of the protein‐triggered gate to deliver therapeutically relevant cargo for controlled cell killing (Figure 4 A). In the assay, pNP2 was inserted into LUVs filled with 3 μm topotecan (Figure 4 B), a clinically used cytotoxic drug active against cervical cancer.^[42]^ The tri‐component vesicles were added to HeLa cervical cancer cells, and thrombin was used to trigger the delivery of the cytotoxic drug to cells (Figure 4 A). Cell viability and death was monitored for 3 d using light microscopy and the WST‐1 colorimetric assay. The results established that the cytotoxic drug (D) was released to lower cell viability only when pNP and thrombin (T) were present (Figure 4 C, Figure S14). After treatment, cell viability was 20±2 % when compared to 95±5 % for cells incubated with buffer (Figure 4 C, LUV/D/pNP + T; term pNP is used instead of pNP2 for clarity and because both molecular gate variants have very similar functional properties).

Controls confirmed that cell viability was minimally affected with either thrombin or pNP (Figure 4 C, Thrombin, pNP). Indeed, thrombin slightly increased viability in line with other studies.^[35]^ Further controls elucidated the effect of each component, and in combination with others, on cell viability. For example, neat drug without encapsulation led to a reduction of cell viability to 42±2 % (Figure 4 C, Drug). Surprisingly, vesicles filled with drug exhibited a similar amount of cytotoxicity (Figure 4 C, LUV/D) indicating that vesicles can fuse with the cell membrane and intracellularly deliver the cytotoxic cargo.[^43^, ^44^] Fusion and uptake may be prevented by modifying the vesicle membrane surface. Indeed, vesicles decorated with the negatively charged membrane‐anchored pNP exhibited reduced cytotoxicity with an increase in cell viability to 55±1 % (Figure 4 C, LUV/D/pNP). By contrast, addition of thrombin to the pNP‐LUVs led to a reduction in cell viability to the previously noted 20±2 % (Figure 4 C, LUV/D/pNP + T). The data support the use of the protein‐triggered valve to deliver therapeutically relevant cargo for controlled cell killing (Figure 4).

## Conclusion

In summary, this study describes the first DNA‐based membrane gate capable of controllably delivering a therapeutic drug to a cellular environment in response to a biologically relevant exogenous trigger. The biomimetic nanodevice is self‐assembled from just seven oligonucleotides and achieves high functional performance by increasing the transport rate 330‐fold upon actuation. Our findings complement other biomimetic DNA nanostructures, including motor activity,[^21^, ^45^, ^46^, ^47^] cellular signal processing,[^48^, ^49^, ^50^] and cytoskeletal support.[^51^, ^52^, ^53^] The gate also advances synthetic biology by facilitating complex function into nanopores.[^54^, ^55^] Previous DNA gates were designed to respond to DNA ligand^[24]^ or elevated temperature^[27]^ but not proteins. Finally, the gate's controllable drug release implies compatibility with potential biomedical applications. Based on its modular design, the nanodevice could be adapted for a range of different protein triggers with applications in biosensing, research, and biomedicine.

## Conflict of interest

The authors declare no conflict of interest.

## Supporting information

As a service to our authors and readers, this journal provides supporting information supplied by the authors. Such materials are peer reviewed and may be re‐organized for online delivery, but are not copy‐edited or typeset. Technical support issues arising from supporting information (other than missing files) should be addressed to the authors.

Supplementary
